# Frequency of glucose-6-phosphate dehydrogenase deficiency in malaria patients from six African countries enrolled in two randomized anti-malarial clinical trials

**DOI:** 10.1186/1475-2875-10-241

**Published:** 2011-08-17

**Authors:** Nick Carter, Allan Pamba, Stephan Duparc, John N Waitumbi

**Affiliations:** 1 ID-MDC Biomedical Data Sciences, GlaxoSmithKline Research and Development, Stockley Park West, Uxbridge, Middlesex, UB11 1BT, UK; 2 Developing Countries & Market Access, GlaxoSmithKline, GSK House CN6 08, 980 Great West Road, Brentford, Middlesex, TW8 9GS, UK; 3 Medicines for Malaria Venture, International Centre Cointrin, 20 Route de Pré-Bois, 1215 Geneva 15, Switzerland; 4 Walter Reed Project/Kenya Medical Research Institute, Kisumu, United Nations Avenue Gigiri, Village Market, Nairobi 00621, Kenya

## Abstract

**Background:**

Glucose-6-phosphate dehydrogenase (G6PD) deficiency is common in populations living in malaria endemic areas. G6PD genotype and phenotype were determined for malaria patients enrolled in the chlorproguanil-dapsone-artesunate (CDA) phase III clinical trial programme.

**Methods:**

Study participants, aged > 1 year, with microscopically confirmed uncomplicated *Plasmodium falciparum *malaria, and haemoglobin ≥ 70 g/L or haematocrit ≥ 25%, were recruited into two clinical trials conducted in six African countries (Burkina Faso, Ghana, Kenya, Nigeria, Tanzania, Mali). G6PD genotype of the three most common African forms, G6PD*B, G6PD*A (A376G), and G6PD*A- (G202A, A542T, G680T and T968C), were determined and used for frequency estimation. G6PD phenotype was assessed qualitatively using the NADPH fluorescence test. Exploratory analyses investigated the effect of G6PD status on baseline haemoglobin concentration, temperature, asexual parasitaemia and anti-malarial efficacy after treatment with CDA 2/2.5/4 mg/kg or chlorproguanil-dapsone 2/2.5 mg/kg (both given once daily for three days) or six-dose artemether-lumefantrine.

**Results:**

Of 2264 malaria patients enrolled, 2045 had G6PD genotype available and comprised the primary analysis population (1018 males, 1027 females). G6PD deficiency prevalence was 9.0% (184/2045; 7.2% [N = 147] male hemizygous plus 1.8% [N = 37] female homozygous), 13.3% (273/2045) of patients were heterozygous females, 77.7% (1588/2045) were G6PD normal. All deficient G6PD*A- genotypes were A376G/G202A. G6PD phenotype was available for 64.5% (1319/2045) of patients: 10.2% (134/1319) were G6PD deficient, 9.6% (127/1319) intermediate, and 80.2% (1058/1319) normal. Phenotype test specificity in detecting hemizygous males was 70.7% (70/99) and 48.0% (12/25) for homozygous females. Logistic regression found no significant effect of G6PD genotype on adjusted mean baseline haemoglobin (p = 0.154), adjusted mean baseline temperature (p = 0.9617), or adjusted log mean baseline parasitaemia (p = 0.365). There was no effect of G6PD genotype (p = 0.490) or phenotype (p = 0.391) on the rate of malaria recrudescence, or reinfection (p = 0.134 and p = 0.354, respectively).

**Conclusions:**

G6PD deficiency is common in African patients with malaria and until a reliable and simple G6PD test is available, the use of 8-aminoquinolines will remain problematic. G6PD status did not impact baseline haemoglobin, parasitaemia or temperature or the outcomes of anti-malarial therapy.

**Trial registration:**

Clinicaltrials.gov: NCT00344006 and NCT00371735.

## Background

Glucose-6-phosphate dehydrogenase (G6PD) is an enzyme in the pentose phosphate pathway (PPP) that plays an important role in protecting cells from oxidative damage by producing NADPH and reduced glutathione. In the erythrocyte, which lacks a nucleus, mitochondria and other organelles, PPP is the only biochemical pathway for generating reducing capacity [1,2]. The *G6PD *gene is highly polymorphic with almost 400 reported variants, conferring varying levels of enzyme activity [2]. *G6PD *is X-linked, and so deficient variants are expressed more commonly in males than in females. Worldwide, an estimated 400 million people are G6PD deficient with the distribution corresponding to areas in which malaria is, or has been, prevalent. This has led to speculation that malaria has been the selection pressure that has favoured the maintenance of this potentially deleterious trait. There is some evidence that hemizygous males may be protected against severe malaria [1,3-5].

Although there are over 400 *G6PD *variants worldwide, in sub-Saharan Africa three variants occur with polymorphic frequencies (> 0.1%); G6PD*B, G6PD*A and G6PD*A-. G6PD*B is the wild type and the most common variant in Africa and worldwide. G6PD*A has a single A→G substitution at nucleotide number 376, though is a normal variant with about 90% of the G6PD*B enzyme activity [6]. G6PD*A- is a deficient variant with about 8-20% of the wild type enzyme activity and, in addition to the A376G mutation that describes the G6PD*A variant, most commonly also involves a G→A substitution at nucleotide 202 [7]. However, G6PD*A- variants with substitutions at 542 G→T, 680 G→T or 968 T→C have also been identified in Africa [3,8].

Individuals with G6PD*A- are normally asymptomatic [1]. However, erythrocyte exposure to oxidative stress causes haemoglobin denaturation, ultimately resulting in haemolysis. Haemolytic anaemia in G6PD-deficient individuals can be triggered by a range of oxidative agents, such as infections and certain foods and drugs, including anti-malarials [9].

This study reports the frequency of G6PD genotypes and phenotypes in malaria patients who participated in two Phase III clinical trials of chlorproguanil-dapsone-artesunate (CDA) [10,11]. The CDA clinical trial programme was terminated because of an increased risk of drug-related haemolysis with CDA in G6PD-deficient compared with G6PD-normal malaria patients [10,11]. The haemolysis was almost certainly because of the dapsone component [10-13]. This paper also reports that G6PD genotype did not influence baseline haemoglobin concentrations, temperature and asexual parasitaemia or the outcomes of anti-malarial efficacy.

## Methods

The clinical data from the CDA trials have been published separately and those reports include more comprehensive descriptions of the conduct of the individual trials [10,11].

### Ethics statement

This study was conducted in accordance with Good Clinical Practices, applicable regulatory requirements, and the Declaration of Helsinki. Approval was obtained from each participating centre's ethics committee or institutional review board and the WHO Special Programme for Research and Training in Tropical Diseases (TDR). An Independent Data Monitoring Committee (IDMC) was convened and planned to conduct two interim safety analyses. The IDMC appointed an independent end-point reviewer (blinded to treatment assignment). Written or oral witnessed informed consent was required from patients or their parent/guardian plus assent from patients who were ≥ 12-18 years old.

### Objectives

This study used genotyping and phenotyping to determine the frequency of G6PD deficiency in malaria patients included in the CDA Phase III clinical trial programme in Africa. Exploratory analyses investigated whether baseline haemoglobin concentration, temperature and asexual parasitaemia were influenced by G6PD genotype. The effect of G6PD status on anti-malarial efficacy was also examined.

### Participants

Patients were screened for inclusion into two clinical trials. Study 005 compared CDA versus artemether-lumefantrine (AL) in malaria patients aged > 1- < 14 years of age and was conducted between June 2006 and August 2007 at 11 centres in five African countries: Bobo-Dioulasso, Burkina Faso; Kintampo, Ghana; Eldoret, Kilifi and Pingilikani, Kenya; Ibadan, Enugu, Jos and Calabar, Nigeria; and Bagamoyo and Kiwangwa, Tanzania [11]. Study 006 compared CDA versus chlorproguanil-dapsone (CPG-DDS) in patients > 1 year old and was conducted between April 2006 to May 2007 at seven health centres in four African countries: Ouagadougou, Burkina Faso; Kumasi, Ghana; Doneguebougou and Banambani, Mali; and Ile-Ife, Jos and Lagos, Nigeria [10].

Across the two trials, eligible subjects included males and females who met the following inclusion criteria: acute uncomplicated microscopically verified *Plasmodium falciparum *malaria (parasite count 2000 to 200,000 μL^-1^); fever at enrollment or history of fever; weight ≥ 7.5 kg; haemoglobin ≥ 70 g/L or haematocrit ≥ 25%; willingness to comply with study procedures.

Subjects were excluded if they had: severe/complicated *P. falciparum *malaria; known G6PD deficiency, methaemoglobin reductase deficiency, haemoglobin M/E (i.e. increased risk of methaemoglobinaemia), or porphyria; neonatal hyperbilirubinaemia; received potentially haemolytic concomitant medication; concomitant infection (including *Plasmodium vivax, Plasmodium ovale *or *Plasmodium malariae*); any underlying disease that could compromise malaria diagnosis or the anti-malarial efficacy assessment; known hypersensitivity/allergy to study treatments or biguanides, sulphones, sulphonamides, or artemisinin derivatives; malnutrition; received recent anti-malarial therapy that may affect the efficacy evaluation or any investigational drug within 30 days or five half-lives (whichever longer); previously participated in the study. A negative pregnancy test was required from women of child-bearing age; breastfeeding mothers were excluded.

### Procedures

During screening, all patients provided a full medical history and underwent a comprehensive clinical examination. Drug treatments were CDA 2/2.5/4 mg/kg, CPG-DDS 2/2.5 mg/kg (both GlaxoSmithKline, Greenford, UK) given once daily for three days (Days 0, 1, 2) or six-dose AL (Novartis Pharma AG, Basel, Switzerland). Randomization was 2:1 CDA versus the comparator. Details of patient assessments are given in the clinical reports of these trials [10,11]. Patients remained hospitalized between Days 0 to 3, with follow up on Days 7, 14, and 28 in both studies, and follow up also at Day 42 in the CDA versus AL study (005); with a home visit from a fieldworker on Days 4, 5, and 6.

### Blinding

G6PD genotype and phenotype were determined by laboratory staff blinded to treatment. Clinical investigators were blinded to drug treatment and G6PD status was not available to clinical investigators until after the trial was complete.

### Parasite assessment

Asexual parasite and gametocyte counts were determined by examination of duplicate Giemsa-stained thick blood slides (10 μL thumb prick) using WHO (2003) methods [14]. Thick blood slides were examined by two microscopists blinded to treatment; a third microscopist read the slides in case of a discrepancy. To differentiate recrudescence from reinfection, two drops of blood collected at enrollment and subsequent date of parasite detection were stored on filter paper for polymerase chain reaction (PCR) analysis of parasite genotype using *msp*-1, *msp*-2 and *glurp *[15,16].

### G6PD genotyping

G6PD genotype analysis was performed at two laboratories: Walter Reed Project/Kenya Medical Research Institute, Kisumu, Kenya and Shoklo Malaria Research Unit, Mae Sot, Thailand. After patient randomization, but before the first dose of study therapy, a 10 μL blood sample was collected (thumb prick) onto pre-printed filter paper. The G6PD genotyping protocol was based on published methods [17-19]. Specific forward primers and reverse primers were used for PCR amplification of a section of *G6PD *gene containing G6PD*B (wild type), the common African mutation G6PD*A (A376G) and the other deficient variant G6PD*A- (G202A, A542T, G680T and T968C). Amplicons were subsequently analysed by restriction fragment length polymorphism (RFLP) and genotypes scored after agarose gel electrophoresis. DNA was extracted using standard methods. PCR was performed using 0.75 U/μL Taq Polymerase, 1.5 mM MgCl2, 200 μM dNTP (mix 10 mM), 2.5 μM of each primer, 10% PCR buffer, and 2.0 μL of DNA. All restriction endonucleases were sourced from New England Biolabs (Ipswich, MA, USA) and used at optimal temperature and digestion period. Genomic DNA was first amplified using primers for A376G, 5'-CCCAGGCCACCCCAGAGGAGA-3' (forward) and 5'-CGGCCCCGGACACGCTCATAG-3' (reverse). Thermocycling was performed at 94°C for 10 min, then 30 cycles at 94°C for 45 sec, 58°C (annealing temperature) for 45 sec, and 72°C for 45 sec, with a final extension at 72°C for 7 min. Amplification products were recovered and PCR fragments incubated at 37°C for 16 h with *Fok*I for identification of the A376G mutation (G6PD*A). All samples positive for A376G were then subjected to PCR amplification using: G202A, 5'-CCACCACTGCCCCTGTGACCT-3' (forward) and 5'-GGCCCTGACACCACCCACCTT-3' (reverse) with annealing at 65°C; G542T, 5'-AGGAGATGTGGTTGGACATCCGG-3' (forward) and 5'-ACTCCCCGAAGAGGGGT-3' (reverse) with annealing at 67°C; G680T, 5'-ACATGTGGCCCCTGCACCAC-3' (forward) and 5'-GTGACTGGCTCTGCCACCCTG-3' (reverse) with annealing at 69°C; and T968C 5'-TCCCTGCACCCCAACTCAAC-3' (forward) and 5'-CCAGTTCTGCCTTGCTGGGC-3' (reverse) with annealing at 65°C. Amplicons were then incubated at 37°C for 16 h with *Nla*III for identification of the G202A mutation, *Acc*III for G542T, *Bst*NI for G680T, and *Nci*I for the T968C mutation. Quality control was maintained by comparing results from the two testing laboratories with discrepant results reanalysed.

### G6PD phenotyping

Pre-dose venous blood samples (2 mL) were collected for G6PD phenotyping, refrigerated to 4°C and transported within seven days to a central laboratory (Synexa, Cape Town, South Africa) for analysis using a commercial NADPH fluorescence test (Trinity Biotech, Wicklow, Ireland). This was a qualitative test designed to distinguish grossly G6PD-deficient samples from those with intermediate/normal enzyme activity. The reaction mixture containing glucose-6-phosphate+NADP (not fluorescent) and spots were made on the filter paper at the beginning (zero-time) and 5, 10, and 20 min after blood incubation with reagent mixture. The spots were visually inspected under long-wavelength (320-420 nm) ultraviolet light. The observed rate of appearance of bright fluorescence is proportional to the blood G6PDH activity; normal samples fluoresce brightly, whereas deficient samples show little or no fluorescence [20].

### Efficacy outcomes

Day-28 parasite recrudescence and reinfection rates, differentiated using PCR genotyping as described above, were used to evaluate whether G6PD genotype or phenotype had an effect on anti-malarial efficacy.

### Statistical methods

Descriptive statistics were used to report demographic and clinical characteristics for patients with G6PD genotype. G6PD genotype and phenotype was presented by country and centre.

All statistical analyses were performed post-hoc. There was no formal sample size calculation for the analyses reported in this paper. Sample size was calculated for clinical endpoints as reported previously [10,11]. Analysis of conformity with the Hardy-Weinberg equilibrium for female gene frequencies was analysed by centre, country and overall. Expected female gene frequencies were calculated based on male genotype prevalence (*A*-, *A *and *B*) and compared with observed values using Chi-squared.

A logistic model was developed to investigate the effect of G6PD genotype on the following baseline variables: haemoglobin concentration, temperature, and asexual parasite count.

The effect of G6PD genotype was evaluated using three categories: normal; heterozygous; or deficient (hemizygous males plus homozygous females). The effect of G6PD genotype on baseline haemoglobin concentration was modelled with terms for age category (< 5, ≥ 5- < 15, ≥ 15 years), weight, centre, sex, temperature and parasitaemia category (four quartiles). The model for the effect of G6PD genotype on temperature had terms for age category, weight, centre, sex, haemoglobin concentration and parasitaemia category. To investigate the effect of G6PD genotype on parasitaemia, parasite densities were initially log transformed with model terms for age category, weight, centre, sex, haemoglobin concentration and temperature.

A logistic model was developed to investigate the effect of G6PD genotype (normal, heterozygous, deficient) on anti-malarial efficacy (recrudescence and reinfection rates) with terms for: anti-malarial treatment, study (CDA vs. AL; CDA vs. CPG-DDS), age category, weight, centre, sex, and parasitaemia category. The same model was used to investigate the effect of G6PD phenotype (normal, intermediate, deficient) on anti-malarial efficacy.

The models were used to calculate adjusted mean values for the variable under investigation and a p value calculated to test any significant effect of model terms using analysis of variance. G6PD genotype was further analysed to compare G6PD normal with heterozygous and G6PD normal with G6PD deficient; 95% confidence intervals were calculated and p values generated using analysis of variance.

## Results

### Patients

**Figure 1:**
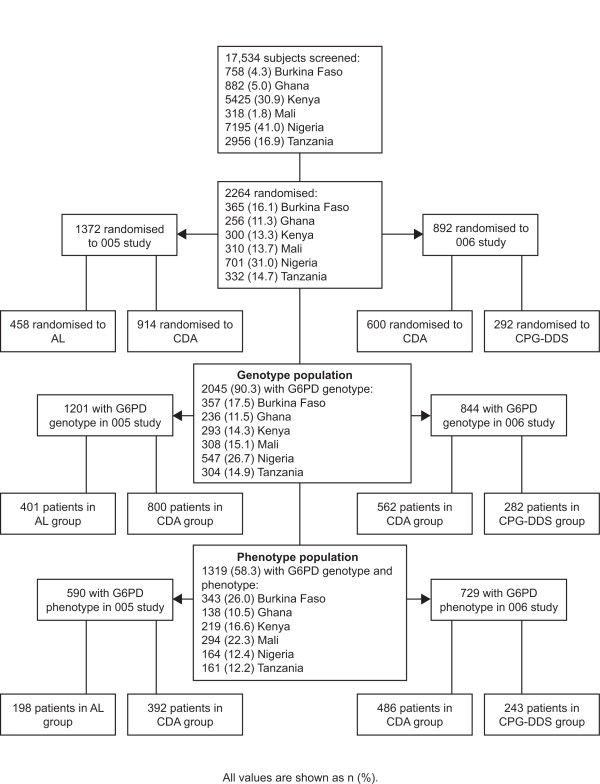
**Trial profile and patient population**.

A summary of the patient population that was screened, randomized and analysed for G6PD status is shown in Figure 1. Across the two studies 2264 malaria patients were enrolled. G6PD genotype and phenotype was available for 2045 and 1319 subjects, respectively, and this comprised the primary analysis population.

### G6PD deficiency prevalence

Table 1 shows G6PD gene frequencies for males (N = 1018) and females (N = 1027) by country and trial centre. There were no G6PD data from Lagos, Nigeria. Of the 2045 patients, 77.7% (1588/2045) were G6PD normal, 13.3% (273/2045) were female heterozygous and 9.0% (184/2045) were G6PD deficient, i.e. 7.2% (147/2045) male hemizygous plus 1.8% (37/2045) female homozygous. All deficient G6PD*A- genotypes were A376G/G202A; there were no instances of A542T, G680T or T968C. Hardy-Weinberg equilibrium was demonstrated for four of 14 centres: Ouagadougou (Burkina Faso), Kilifi (Kenya), Banambani (Mali) and Kiwangwa (Tanzania) (Table 1).

**Table 1:** Frequency of G6PD genotypes in malaria patients from 17 centres in six African countries

Country	Centre	Male N	*A-*	*A*	*B*	Female N	*A-A-*	*AA-*	*BA-*	*AA*	*BB*	*BA*	HW* p
BF	Bobo-Dioulasso	1	0	1 (100)	0	4	0	0	2 (50.0)	0	1 (25.0)	1 (25.0)	NC
	Ouagadougou	174	27 (15.5)	48 (27.6)	99 (56.9)	178	6 (3.4)	15 (8.4)	33 (18.5)	18 (10.1)	54 (30.3)	52 (29.2)	0.742
	Total	175	27 (15.4)	49 (28.0)	99 (56.6)	182	6 (3.3)	15 (8.2)	35 (19.2)	18 (9.9)	55 (30.2)	53 (29.1)	0.771
Ghana	Kintampo	92	16 (17.4)	52 (56.5)	24 (26.1)	87	8 (9.2)	10 (11.5)	21 (24.1)	9 (10.3)	22 (25.3)	17 (19.5)	0.003
	Kumasi	19	2 (10.5)	4 (21.1)	13 (68.4)	38	1 (2.6)	1 (2.6)	5 (13.2)	6 (15.8)	13 (34.2)	12 (31.6)	0.019
	Total	111	18 (16.2)	28 (25.2)	65 (58.6)	125	9 (7.2)	11 (8.8)	26 (20.8)	15 (12.0)	35 (28.0)	29 (23.2)	0.001
Kenya	Eldoret	77	15 (19.5)	22 (28.6)	40 (51.9)	72	2 (2.8)	2 (2.8)	10 (13.9)	1 (1.4)	42 (58.3)	15 (20.8)	< 0.001
	Kilifi	41	8 (19.5)	10 (24.4)	23 (56.1)	33	2 (6.1)	3 (9.1)	8 (24.2)	1 (3.0)	10 (30.3)	9 (27.3)	0.961
	Pingilikani	39	5 (12.8)	7 (17.9)	27 (69.2)	31	1 (3.2)	7 (22.6)	5 (16.1)	1 (3.2)	11 (35.5)	6 (19.4)	< 0.001
	Total	157	28 (17.8)	39 (24.8)	90 (57.3)	136	5 (3.7)	12 (8.8)	23 (16.9)	3 (2.2)	63 (46.3)	30 (22.1)	0.016
Mali	Doneguebougou	114	10 (8.8)	41 (36.0)	63 (55.3)	102	2 (2.0)	4 (3.9)	20 (19.6)	14 (13.7)	39 (38.2)	23 (22.5)	< 0.001
	Banambani	48	7 (14.6)	13 (27.1)	28 (58.3)	44	1 (2.3)	1 (2.3)	7 (15.9)	2 (4.5)	23 (52.3)	10 (22.7)	0.176
	Total	162	17 (10.5)	54 (33.3)	91 (56.2)	146	3 (2.1)	5 (3.4)	27 (18.5)	16 (11.0)	62 (42.5)	33 (22.6)	< 0.001
Nigeria	Calabar	76	11 (14.5)	11 (14.5)	54 (71.1)	69	0	7 (10.1)	13 (18.8)	5 (7.2)	33 (47.8)	11 (15.9)	0.005
	Enugu	121	17 (14.1)	32 (26.4)	72 (59.5)	98	5 (5.1)	11 (11.2)	23 (23.5)	1 (1.0)	41 (41.8)	17 (17.3)	0.001
	Ibadan	26	2 (7.7)	2 (7.7)	22 (84.6)	30	0	3 (10.0)	4 (13.3)	3 (10.0)	15 (50.0)	5 (16.7)	< 0.001
	Ile Ife	49	7 (14.3)	14 (28.6)	28 (57.1)	57	5 (8.8)	4 (7.0)	14 (24.6)	4 (7.0)	20 (35.1)	10 (17.5)	0.002
	Jos	11	0	4 (36.4)	7 (63.6)	10	0	2 (20.0)	1 (10.0)	3 (30.0)	2 (20.0)	2 (20.0)	NC
	Total	283	37 (13.1)	63 (22.3)	183 (64.7)	264	10 (3.8)	27 (10.2)	55 (20.8)	16 (6.1)	111 (42.0)	45 (17.0)	< 0.001
Tanzania	Bagamoyo	19	4 (21.1)	4 (21.1)	11 (57.9)	23	0	0	0	5 (21.7)	13 (56.5)	5 (21.7)	< 0.001
	Kiwangwa	111	16 (14.4)	18 (16.2)	77 (69.4)	151	4 (2.6)	11 (7.3)	26 (17.2)	3 (2.0)	60 (39.7)	47 (31.1)	0.063
	Total	130	20 (15.4)	22 (16.9)	88 (67.7)	174	4 (2.3)	11 (6.3)	26 (14.9)	8 (4.6)	73 (42.0)	52 (29.9)	0.094
Total		1018	147 (14.4)	283 (27.8)	588 (57.8)	1027	37 (3.6)	81 (7.9)	192 (18.7)	76 (7.4)	399 (38.8)	242 (23.5)	< 0.001

Data shown as n (%) BF, Burkina Faso; NC, not calculated. G6PD genotype: male hemizygous = *A-*; male normal = *A *or *B*; female homozygous = *A-/A-*; female heterozygous = *A/A- *or *B/A-*; and female normal = *A/A, B/B *or *B/A*

*p values < 0.05 indicate significant differences from predicted *G6PD *gene frequencies in females based on male gene frequencies for A-, A and B alleles using the Hardy-Weinberg equation (Chi-squared)

G6PD phenotype results are shown in Table 2. Phenotype was available for 64.5% (1319/2045) of patients. Overall, 10.2% (134/1319) of patients were phenotypically G6PD deficient, 9.6% (127/1319) intermediate, and 80.2% (1058/1319) normal (Table 2). Of the G6PD*A- hemizygous males that had phenotype data available, 70/99 (70.7%) were phenotypically G6PD deficient and 29/99 (29.3%) had intermediate/normal phenotype (Table 3). Of the G6PD*A- homozygous females, 12/25 (48.0%) had deficient phenotype and 13/25 (52.0%) had normal/intermediate phenotype (Table 3). Likewise, 39/1021 (3.1%) patients of normal G6PD genotype (males A or B, females AA, AB or BB) were classified as G6PD deficient and 68/1021 (6.7%) as intermediate, illustrating the fluidity of G6PD measurements. As expected, female heterozygous displayed a wide range of G6PD activity.

**Table 2:** Frequency of G6PD phenotype by centre and country

Country	Centre	N*	Deficient	Intermediate	Normal
BF	Bobo-Dioulasso	0	0	0	0
	Ouagadougou	343	29 (8.5)	37 (10.8)	277 (80.8)
	Total	343	29 (8.5)	37 (10.8)	277 (80.8)
Ghana	Kintampo	118	8 (6.8)	15 (12.7)	95 (80.5)
	Kumasi	20	1 (5.0)	0	19 (95.0)
	Total	138	9 (6.5)	15 (10.9)	114 (82.6)
Kenya	Eldoret	90	15 (16.7)	4 (4.4)1	71 (78.9)
	Kilifi	67	7 (10.4)	7 (10.4)	53 (79.1)
	Pingilikani	62	4 (6.5)	3 (4.8)	55 (88.7)
	Total	219	26 (11.9)	14 (6.4)	179 (81.7)
Mali	Doneguebougou	205	17 (8.3)	20 (9.8)	168 (82.0)
	Banambani	89	10 (11.2)	11 (12.4)	68 (76.4)
	Total	294	27 (9.2)	31 (10.5)	236 (80.3)
Nigeria	Calabar	21	5 (23.8)	2 (9.5)	14 (66.7)
	Enugu	71	6 (8.5)	10 (14.1)	55 (77.5)
	Ibadan	0	0	0	0
	Ile Ife	72	7 (9.7)	4 (5.6)	61 (84.7)
	Jos	0	0	0	0
	Total	164	18 (11.0)	16 (9.8)	130 (79.3)
Tanzania	Bagamoyo	10	2 (20.0)	0	8 (80.0)
	Kiwangwa	151	23 (15.2)	14 (9.3)	114 (75.5)
	Total	161	25 (15.5)	14 (8.7)	122 (75.8)
Total		1319	134 (10.2)	127 (9.6)	1058 (80.2)

Frequencies are shown as n (%)

*N is the number of patients with genotype and phenotype data.

**Table 3:** Relationship between phenotype and genotype testing

G6PD genotype	G6PD phenotype	n/N (%)
Male *A-*	Deficient	70/99 (70.7)
	Intermediate	19/99 (19.2)
	Normal	10/99 (10.1)
Male *A *or *B*	Deficient	25/575 (4.3)
	Intermediate	28/575 (4.9)
	Normal	522/575 (90.8)
Female *A-/A-*	Deficient	12/25 (48.0)
	Intermediate	7/25 (28.0)
	Normal	6/25 (24.0)
Female *A-/A *or A-/B**	Deficient	13/174 (7.5)
	Intermediate	33/174 (19.0)
	Normal	128/174 (73.6)
Female *AA, AB *or *BB*	Deficient	14/446 (3.1)
	Intermediate	40/446 (9.0)
	Normal	392/446 (87.9)

G6PD genotype: male hemizygous = *A-*; male normal = *A *or *B*; female homozygous = *A-/A-*; female heterozygous = *A/A- *or *B/A-*; and female normal = *A/A, B/B *or *B/A*

### G6PD genotype effect on baseline variables

**Figure 2:**
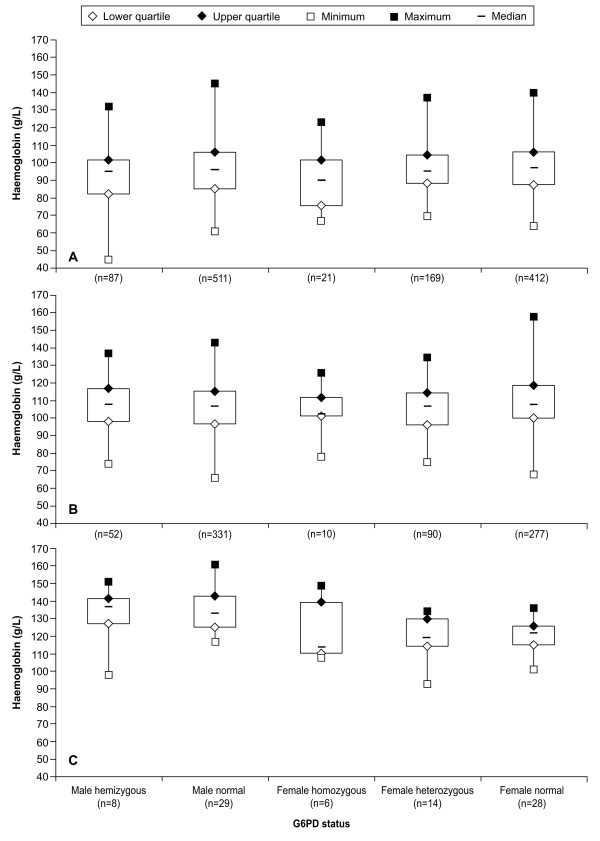
**Baseline haemoglobin by G6PD genotype for patients aged: A, 1- < 5 years; B, 5- < 15 years; and C, ≥ 15 years**. G6PD genotype: male hemizygous = *A-*; male normal = *A *or *B*; female homozygous = *A-/A-*; female heterozygous = *A/A- *or *B/A-*; and female normal = *A/A, B/B *or *B/A*.

Baseline demographic and clinical characteristics of the study participants are summarized in Table 4. Figure 2 shows baseline haemoglobin categorized by G6PD genotype and age. Logistic regression found no significant effect of G6PD genotype on adjusted mean baseline haemoglobin (p = 0.154). Comparison between G6PD normal and heterozygous (p = 0.693), or between G6PD normal and deficient genotype (p = 0.0559) showed no difference in adjusted mean baseline haemoglobin. Note that this population was selected to have a baseline haemoglobin ≥ 70 g/L and this may have biased the results.

**Table 4:** Baseline demographic and clinical data by G6PD genotype

Characteristic*	Male hemizygous (n = 147)	Male normal (n = 871)	Female homozygous (n = 37)	Female heterozygous (n = 273)	Female normal (n = 717)	All patients (n = 2045)
Age, years [range]	5.8 (8.0) [1-70]	5.1 (5.5) [1-59]	7.9 (10.0) [1-42]	5.6 (7.8) [1-72]	5.6 (6.5) [1-72]	5.44 (6.5) [1-72]
1- < 5, n (%)	87 (59)	511 (59)	21 (57)	169 (62)	412 (57)	1200 (59)
5- < 15, n (%)	52 (35)	331 (38)	10 (27)	90 (33)	277 (39)	760 (37)
≥ 15, n (%)	8 (5)	29 (3)	6 (16)	14 (5)	28 (4)	85 (4)
Weight, kg, mean	18.8 (11.7)	17.9 (10.5)	23.1 (21.9)	18.0 (12.2)	18.3 (11.0)	18.2 (11.3)
Parasitaemia, μL^-1^, geometric mean [range]	25990 [1484-705600]	26331 [216-323361]	18602 [1026-211546]	22498 [0-303400]	27213 [0-389415]	25895 [0-705600]
Temperature, °C	37.8 (1.0)	37.9 (1.0)	37.9 (1.0)	37.8 (1.0)	37.9 (1.0)	37.9 (1.0)
Fever, n (%)	81 (55.1)	552 (63.4)	24 (65.0)	165 (60.0)	459 (64.0)	1281 (62.6)
Haemoglobin, g/L*	100.3 (18.3)	101.1 (16.8)	99.7 (19.2)	100.6 (14.2)	102.7 (15.6)	101.5 (16.2)
1 to < 5 years	93.2 (15.2)	95.6 (14.6)	91.0 (16.5)	96.1 (12.6)	97.3 (14.6)	96.0 (14.5)
5 to < 15 years	107.2 (15.2)	106.6 (14.8)	104.4 (12.5)	105.8 (13.2)	108.9 (13.9)	107.3 (14.3)
≥ 15 years	132.5 (16.5)	135.7 (12.2)	122.5 (17.5)	119.9 (11.9)	120.5 (9.8)	126.9 (14.0)
Haematocrit, L	0.30 (0.05)	0.30 (0.05)	0.31 (0.05)	0.31 (0.04)	0.31 (0.04)	0.31 (0.05)
RBC count x10^12^/L	4.0 (0.7)	4.2 (0.6)	4.0 (0.6)	4.0 (0.5)	4.1 (0.6)	4.1 (0.62)
Platelet count x10^9^/L	191.4 (86.5)	196.7 (111.6)	206.1 (125.2)	186.9 (96.4)	198.1 (128.0)	195.7 (114.5)
WBC count x10^9^/L	9.6 (4.3)	9.5 (4.1)	8.5 (4.2)	9.5 (3.9)	9.4 (4.0)	9.5 (4.0)
Treatment n (%)						
CDA 005	64 (43.5)	324 (37.2)	16 (43.2)	123 (45.1)	273 (38.1)	800 (39.1)
CDA 006	38 (25.9)	250 (28.7)	6 (16.2)	70 (25.6)	198 (27.6)	562 (27.5)
AL	30 (20.4)	185 (21.2)	6 (16.2)	43 (15.8)	137 (19.1)	401 (19.6)
CPG-DDS	15 (10.2)	112 (12.9)	9 (24.3)	37 (13.6)	109 (15.2)	282 (13.8)

All values are mean (SD) [range] unless otherwise indicated. CDA, chlorproguanil-dapsone-artesunate; AL, artemether-lumefantrine; CPG-DDS, chlorproguanil-dapsone; RBC, red blood cell; WBC, white blood cell. G6PD genotype: male hemizygous = *A-*; male normal = *A *or *B*; female homozygous = *A-/A-*; female heterozygous = *A/A- *or *B/A-*; and female normal = *A/A, B/B *or *B/A*

*There were 23 patients with baseline haemoglobin of < 70 g/L (range 45-69 g/L, mean 65 g/L). This occurred because at some sites screening haemoglobin level was determined using a rapid test (Haemacue, Downfield, UK), with subsequent checking using a coulter counter the results of which are reported here.

**Figure 3:**
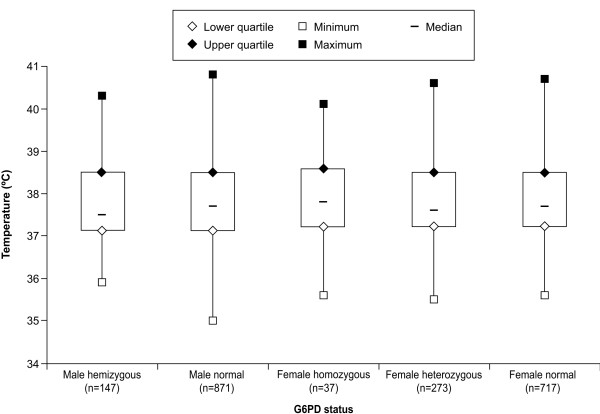
**Baseline temperature by G6PD genotype**. G6PD genotype: male hemizygous = *A-*; male normal = *A *or *B*; female homozygous = *A-/A-*; female heterozygous = *A/A- *or *B/A-*; and female normal = *A/A, B/B *or *B/A*.

**Figure 4:**
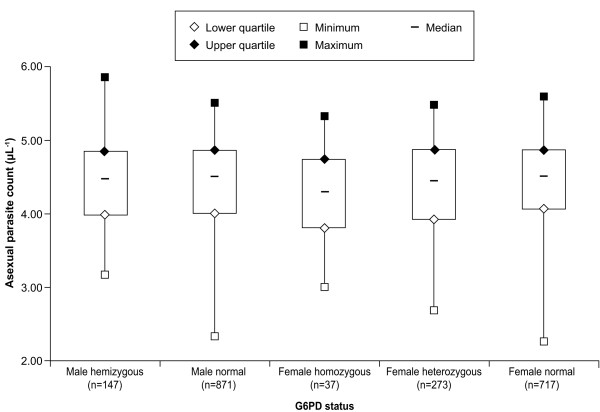
**Baseline parasitaemia (Log_10_) by G6PD genotype**. G6PD genotype: male hemizygous = *A-*; male normal = *A *or *B*; female homozygous = *A-/A-*; female heterozygous = *A/A- *or *B/A-*; and female normal = *A/A, B/B *or *B/A*.

Figures 3 and 4 show baseline temperature and parasitaemia categorized by G6PD genotype, respectively. Logistic regression showed no significant effect of G6PD genotype on adjusted mean baseline temperature (p = 0.9617). Comparisons of adjusted mean baseline temperature between G6PD normal and heterozygous (p = 0.784) or G6PD normal and deficient genotype (p = 0.969) found no significant differences. G6PD genotype had no significant effect on adjusted log mean baseline parasitaemia (p = 0.365). There was no difference in adjusted log mean parasitaemia between G6PD normal versus heterozygous (p = 0.181) or G6PD normal versus deficient genotype (p = 0.693).

### G6PD genotype and anti-malarial efficacy

Table 5 shows recrudescence and reinfection rates by genotype and phenotype. There were 104/2045 (5.1%) patients with parasite recrudescence during the CDA clinical trials. Logistic modelling found no effect of G6PD genotype (p = 0.490) or phenotype (p = 0.391) on recrudescence rate. There was also no significant difference in recrudescence rate between normal and heterozygous G6PD genotypes (odds ratio [OR] 0.7; 95%CI 0.4, 1.4; p = 0.315) or normal and deficient genotypes (OR 1.2; 95%CI 0.6, 2.4; p = 0.561).

**Table 5:** Malaria recrudescence and reinfection rates at Day 28 by G6PD genotype and phenotype

G6PD status	N	Recrudescence n (%)	Reinfection n (%)
Genotype*			
Deficient	184	11 (6.0)	49 (26.6)
Heterozygous	273	11 (4.0)	71 (26.0)
Normal	1588	82 (4.0)	488 (30.7)
Total	2045	104 (5.1)	608 (29.7)
Phenotype**			
Deficient	146	9 (6.2)	39 (26.7)
Intermediate	137	3 (2.2)	39 (28.5)
Normal	1096	62 (5.7)	391 (35.7)
Total	1379	74 (5.4)	469 (34.0)

*G6PD genotype: male hemizygous = *A-*; male normal = *A *or *B*; female homozygous = *A-/A-*; female heterozygous = *A/A- *or *B/A-*; and female normal = *A/A, B/B *or *B/A*

**Analysis performed for patients with G6PD phenotype with or without genotype available.

Reinfection with falciparum malaria occurred in 608/2045 (29.7%) patients overall in the study. Based on the raw data (Table 5), there was a trend for lower reinfection rates in patients that had G6PD-deficient (26.6%) or heterozygous genotypes (26.0%) versus normal genotype (30.7%). Also, patients with a deficient phenotype had a lower reinfection rate (26.7%) than those with a normal phenotype (35.7%). However, within the logistic model these relationships failed to reach statistical significance overall: for genotype p = 0.134 and for phenotype p = 0.354. The difference in reinfection rate also failed to reach statistical significance between normal and heterozygous G6PD genotypes (OR 0.8; 95%CI 0.5, 1.1; p = 0.105) or normal and deficient genotypes (OR 0.8; 95%CI 0.5, 1.1; p = 0.203).

## Discussion

The primary objective of this study was to describe the prevalence of G6PD genotypes in African malaria patients who participated in two CDA Phase III clinical trials. In this study, G6PD*A- prevalence varied between trial centres and between countries (Table 1). Where comparable data exists for each country, the prevalence of G6PD deficiency in the current study was lower compared with previous reports from Burkina Faso (31.0%) [21], and Nigeria (21.6%, 23.9% and 24.2%) [22-24], but higher than reported for Ghana (8.5%) [25]. A study in Mali of children with uncomplicated or complicated malaria, reported a G6PD*A- prevalence of 12.9% [5]; about the same as reported here (10.5%). There are no previous reports for G6PD genotype prevalence using DNA analysis for Kenya or Tanzania for comparison.

The current study selected for particular malaria patients, so it is not surprising that G6PD gene frequencies are divergent from those reported for randomly selected healthy individuals in the general population. Notably, patients with known G6PD deficiency or neonatal hyperbilirubinaemia were excluded. Unfortunately, data are not available on how many patients were excluded from the clinical trials because of known G6PD deficiency. However, anecdotally, there were probably very few as the regions studied were naïve to G6PD testing. Conversely, G6PD prevalence data from a general population cannot necessarily be used to estimate the proportion of G6PD-deficient individuals that will be enrolled into an anti-malarial clinical trial. From the postulated evolution of G6PD deficiency, it is possible that the frequency of malaria is lower in patients with G6PD deficiency, though a protective effect against uncomplicated malaria has not been conclusively demonstrated.

In this study, all the G6PD*A- mutations involved A376G/G202A, confirming this variant as the most common in Africa. The A542T, G680T or T968C G6PD*A- mutations were not detected in the malaria patients included in this study. Data on the frequency of these mutations are sparse in Africa. The 968C mutation has been reported as the most common G6PD*A- allele in The Gambia [3] and in the Sereer ethnic group from Senegal [8]. Given the selected population in this study it cannot be confirmed whether any of these mutations are not present in the countries studied. The limitations of the assay regarding other possible G6PD-deficient mutations that were not tested for in this study should also be considered. Further genotyping studies are required to determine the frequency of these mutations in the general population and specific ethnic groups.

The Hardy-Weinberg equation describes a population in which both allele and genotype frequencies do not change, meaning they are in equilibrium. Using this equation, ten centres had female gene frequencies significantly different to those predicted from male gene frequencies (Table 1). The sampling methods in the studied population (as determined by the study inclusion/exclusion criteria) may not have enrolled the different genotypes at the same frequencies that they occur in the general population. Also, the sample size was not large enough in some centres to confirm or reject the hypothesis of genetic equilibrium. Other explanations include changes in selection pressure, such as from malaria control or anti-malarial treatment, or population mixing. Evidence from Taiwan indicates that recent immigration can cause changes in relative G6PD gene frequencies between males and females independent of the overall incidence of G6PD deficiency [26]. This may be relevant for some of the centres in this study; for example, Eldoret is one of the fastest growing towns in Kenya, and with 250 different ethnic groups in the country, it would be unlikely if genetic equilibrium for G6PD genotypes was maintained. However, it is difficult to separate the complex reasons for the divergence from Hardy-Weinberg equilibrium and further specific investigations would be required. There is also a methodological explanation. In many studies on G6PD-deficiency variant prevalence, only the frequency of the *A- *allele is known and this alone is used to derive predicted female genotype frequencies for G6PD deficient, heterozygous and normal; thus, only the *A- *allele is tested for equilibrium. In this study, the frequencies of the *A-, A *and *B *alleles in males were used to calculate predicted frequencies of the female genotypes, *A-A-, AA-, BA-, AA, BB *and *BA*, which is a strict application of the Hardy-Weinberg equation and so less likely to show conformance unless all G6PD alleles and genotypes are in equilibrium.

G6PD genotyping has limitations, for instance, it is not practical as a routine test in a clinical setting. Also, because the gene is highly polymorphic (more than 400 reported variants), unusual but clinically important variants can be missed [2]. Phenotyping using the NADPH fluorescence spot test is an alternative test as it detects deficiency irrespective of underlying gene mutations (Table 2). The biggest limitation of G6PD phenotyping is its inability to conclusively identify heterozygous females because their G6PD levels can range from near normal to deficient. As shown in Table 3, 73.6% (128/174) of female heterozygous patients were classified as 'normal' by G6PD phenotyping. In the CDA trials, heterozygous females appeared to have no greater risk for clinically significant haemolysis than normal females (haemoglobin decrease of ≥ 40 g/L or ≥ 40% versus baseline or haemoglobin < 50 g/L or blood transfusion) [10,11]. However, this cannot be assumed for all drugs.

As shown in Table 3, the specificity of phenotype testing for identifying G6PD*A- was 66.1% (82/124), i.e. 33.9% (42/124) of G6PD*A- patients were not identified by phenotype. This misclassification is probably attributable to reticulocyte rebound subsequent to haemolytic crisis, or recovery from malaria [9]. In patients with malaria, higher than expected G6PD enzyme levels can occur in individuals with G6PD-deficient genotypes because the increased erythrocyte replacement rate results in a younger erythrocyte population and newly formed erythrocytes have a greater capacity for G6PD production [9]. However, for unknown reasons, the correlation between deficient phenotype and G6PD*A- genotype was better for hemizygous males, specificity 70.7% (70/99), than for homozygous females, specificity 48.0% (12/25). This has also been seen in a recent paper where 83.3% (30/36) of hemizygous males were phenotypically deficient whereas specificity was only 60.0% (3/5) in homozygous females, though numbers in the study were very low compared with the current dataset [27]. In males and females with normal G6PD genotype (*A, B, AA *or *AB*), 4.3% (25/575) and 3.1% (14/446), respectively, were found to be phenotypically deficient. It is possible that these individuals might have had G6PD mutations at loci other than those examined.

In the context of this trial, using only phenotype data would have significantly undermined interpretation of the drug-induced G6PD-related haemolytic effect of CDA as about a third of G6PD*A- patients would be expected to have had a clinically important haemolysis [10,11]. Because of this, and the limitation in identifying heterozygous females, G6PD genotyping will continue to be important, particularly in a research situation. In the wider context, it has been suggested that phenotype could be used to exclude G6PD*A- patients from receiving potentially harmful treatments. It is possible to calculate the number of G6PD*A- patients that would have been inadvertently treated after phenotype screening (Table 3). Excluding the 134 patients who had a deficient phenotype would leave a total population of 1185 exposed to treatment, 42 (3.5%) of whom were of G6PD*A- genotype. Excluding patients with a deficient or intermediate phenotype (n = 261), leaves a total population of 1058, of whom 16 (1.5%) had a G6PD*A- genotype. The number of G6PD*A- patients misclassified by phenotype appears small (1.5%), i.e. about five patients would have had significant hemolysis that would have been avoidable with genotyping. However, where alternative therapies exist, in the clinical setting it would be unethical to expose even a small proportion of patients with a G6PD-deficient genotype to potentially life-threatening haemolysis, particularly where patient follow up is limited.

Across all patients, phenotype correlated reasonably well with the occurrence of significant hemolysis. Of the 878 patients with phenotype data, significant hemolysis occurred in 21.1% (19/90) of those patients who were phenotypically deficient, in 5.6% (5/90) of those who were intermediate and in 1.1% (8/698) of those with normal phenotype. Of the 41/124 (33.0%) G6PD*A- patients treated with CDA who had clinically important haemolysis, 23 had phenotype data available. Of these, 17/23 (73.9%) were phenotypically deficient, 4/23 (17.4%) had intermediate phenotype and 2/23 (8.7%) had normal phenotype.

The study design excluded patients with haemoglobin concentrations ≤ 70 g/L and this consideration may have affected the study results. There were no independent effects of G6PD genotype on baseline haemoglobin, temperature, or malaria parasitaemia in the study population (Table 4, Figures 2, 3 and 4). Thus, judged by these parameters, G6PD-deficient malaria patients appeared to be no different from other malaria patients at presentation. These results are consistent with a study in Tanzania in malaria patients that showed no effect of G6PD genotype on haemoglobin concentration or parasite density [28]. Also, in Nigerian subjects with no or asymptomatic malaria, G6PD deficiency had no significant effect on haemoglobin levels, though red blood cell concentrations were higher in G6PD-normal subjects versus those who were G6PD deficient [23].

As shown in Table 5, there was a trend for patients with G6PD heterozygous or deficient genotypes or G6PD deficient phenotype to have lower unadjusted reinfection rates. However, after adjustment for baseline factors, including drug treatment, no effect of G6PD genotype or phenotype on recrudescence or reinfection rates was found. A recent study found no effect of G6PD deficiency on *P. falciparum *clearance after treatment with artemisinin-based combination therapy [29]. However, there is some evidence that G6PD deficiency may reduce the number of parasite strains carried by individuals in transmission zones/seasons [30].

There are limitations in performing post-hoc statistical analysis on the effect of G6PD status on baseline variables and efficacy as was done in this study. Logistic modelling also has limitations; inclusion of the variables for adjustment is somewhat subjective and can never be comprehensive. Therefore, the conclusions that can be drawn from this study should be viewed as exploratory and require verification in prospective studies.

## Conclusions

The G6PD*A- deficient genotype was relatively common among the diverse African populations included in this study. This represents a significant risk for adverse haemolytic events after treatment with drug therapies that have the potential to induce oxidative stress. G6PD genotyping and phenotyping should therefore be a requisite in clinical trials evaluating the safety and efficacy of such drugs. The G6PD prevalence data presented here are relevant for the design of such anti-malarial clinical trials. Lastly, there were no clinical differences between G6PD-deficient and -normal patients with malaria enrolled into the CDA clinical trials.

## Competing interests

Nick Carter and Allan Pamba are employees of GlaxoSmithKline. Stephan Duparc is an employee of the Medicines for Malaria Venture.

## Author contributions

All authors contributed to the study design. Genotype testing and scoring was performed in JW's laboratory. NC conducted the statistical analysis. All authors contributed to the preparation of the manuscript and approved the final version.
